# ACC deaminase producing rhizobacterium *Enterobacter cloacae* ZNP-4 enhance abiotic stress tolerance in wheat plant

**DOI:** 10.1371/journal.pone.0267127

**Published:** 2022-05-06

**Authors:** Rajnish Prakash Singh, Dev Mani Pandey, Prabhat Nath Jha, Ying Ma

**Affiliations:** 1 Department of Bioengineering and Biotechnology, Birla Institute of Technology, Mesra, Ranchi, Jharkhand, India; 2 Department of Biological Sciences, Birla Institute of Technology & Science, Pilani, Rajasthan; 3 College of Resources and Environment, Southwest University, Chongqing, China; United Arab Emirates University, UNITED ARAB EMIRATES

## Abstract

Plant growth promoting rhizobacterium (PGPR) designated as ZNP-4, isolated from the rhizosphere of *Ziziphus nummularia*, was identified as *Enterobacter cloacae* following 16S rRNA sequence analysis. The isolated strain exhibited various plant growth promoting (PGP) traits. The 1-aminocyclopropane-1-carboxylic acid deaminase (ACCD) activity was evaluated under diverse physiological conditions that could be useful for minimizing the abiotic stress-induced inhibitory effects on wheat plants. The strain showed resistance to salt (NaCl) and metal (ZnSO_4_) stress. The effect of *E*. *cloacae* ZNP-4 on the augmentation of plant growth was studied under salinity stress of 150 mM (T1 treatment) & 200 mM (T2 treatment) NaCl. The inoculation of strain ZNP-4 significantly improved the various growth parameters of wheat plant such as shoot length (41%), root length (31%), fresh weight (28%), dry weight (29%), photosynthetic pigments chlorophyll a (62%) and chlorophyll b (34%). Additionally, the strain was found to be efficient for minimizing the imposed Zn stress in terms of improving plant growth, biomass and photosynthetic pigments in pots containing different levels of metal stress of 150 mg kg^-1^ (treatment T1) and 250 mg kg^-1^ (treatment T2). Isolate ZNP-4 also improved the proline content and decreased malondialdehyde (MDA) level under both salinity and metal stress, therefore maintaining the membrane integrity. Furthermore, bacterial inoculation increased the activities of antioxidative enzymes such as superoxide dismutase (SOD), catalase (CAT), and peroxidase (POX). The positive effects of PGPR occurred concurrently with the decrease in abiotic stress-induced reactive oxygen species (ROS) molecules such as hydrogen peroxide (H_2_O_2_) and superoxide (O_2_^-^) contents. Overall, the observed results indicate that use of bacteria with such beneficial traits could be used as bio-fertilizers for many crops growing under stress conditions.

## Introduction

In developing countries, crop production is severely affected by the adverse effects of abiotic stressors. Among abiotic stressors, soil salinity is a major constraint for the cultivation of crops across the globe. It is estimated that approximately 1.5 billion hectares of agricultural land are adversely affected by soil salinity [1]. Additionally, it has been estimated that more than 50% of arable land would be severely affected by salinity by the year 2050 [2]. The global increase in soil salinization constitutes a major environmental threat for crop yield and production [3]. Apart from salinity, the presence of various heavy metals in soils imposes toxic effects on plants and also alters the microbial community in soil [4, 5]. The accumulation of heavy metals in soils severely affects the soil texture, its nutrient contents, and also hampers plant productivity by affecting various plant physiological and molecular activities [6]. This metal-imposed toxic effect is an important responsive factor regarding food crisis especially in heavily populated countries like India [7].

Salinity in soils imposes the osmotic imbalance by lowering the soil water potential. The accumulation of water-soluble Na^+^ and Cl^-^ ions generates toxic damages as well as hinders the absorption of essential nutrients like Ca^2+^ and K^+^ [8]. Strategies to alleviate salinity stress could involve the development of salt-resistant varieties, reducing the accumulation of salt through proper cultivation and cropping system etc. [9]. However, all these practices are costly and time consuming. The association between PGPR and their host plants are thought to be ancient. However, recently microbes-mediated plant stress management has been widely accepted as a cost-effective procedure for stress amelioration in plants and their role in improving growth and productivity has been well established [10, 11]. The use of such microorganisms termed as “biofertilizers/or bioinoculants” in agriculture has received the attention of scientists throughout the world [12]. Similarly, numerous plant-associated microbes exert favourable effects on plants under heavy metal stress through multifarious mechanisms [13, 14].

PGPR comprises a heterogeneous group of microorganisms residing in the rhizosphere and enhance plant growth directly through providing mineral nutrition, phytohormone production or indirectly through protection of plants from pathogenic microorganisms [11]. In addition, certain PGPR can also modulate the defense machinery to protect plants from abiotic stressors termed as ‘induced systemic tolerance’ (IST) [15].

Many of the rhizosphere bacteria produce ACCD, which reduces the level of ‘stress ethylene’ in their associated plants by degrading ACC to ammonia and α-ketobutyrate, thereby minimizing the substrate availability for ethylene generation. The previous study has shown that micro-organisms with ACCD activity >20 nmol α-ketobutyrate mg^-1^ h^-1^ are sufficient to enhance plant growth under stress conditions [16]. The effectiveness of ACCD-producing bacteria under various abiotic stressors has been proven by many researchers [17–20]. PGPR with phosphatase activity solubilize the insoluble forms of phosphorus to their soluble form and provides to the plants under adverse environmental conditions. Indole -3-acetic acid (IAA)-producing bacteria enhance the lateral root development for the acquisition of nutrients from soils [21]. Additionally, PGPR increase the root adhering soil, stabilize the soil aggregates for providing better soil structure and protect plants from salinity-induced physiological drought conditions [22]. Similarly, the production of exopolysaccharides by PGPR has shown profound effects in the rhizosheath formation to protect plant from desiccation, maintain the microbial aggregation, its surface attachment and bioremediation [23].

The increased abiotic stress enhance the formation of reactive oxygen species (ROS) such as superoxide (O_2_^-^), hydrogen peroxide (H_2_O_2_) and hydroxyl radicals (OH^-^), which leads to lipid peroxidation, membrane deterioration, metabolic and structural dysfunctions, further leading to cell death [24]. These ROS molecules exert oxidative damages at the cellular level due to their poor detoxification. Salinity leads to the disbalance of ions in the cytosol of plants. This might be attributed to the competition of Na^+^ and Cl^−^ with other nutrients such as K^+^ and Ca^2+^ [25]. The enhancement of activities of antioxidant enzymes such as SOD, CAT and POX are involved in scavenging toxic ROS and averting stress-induced damages [24]. Inoculation of lettuce (*Lactuca stiva* L.) with PGPR *Pseudomonas mendocina* alleviated the oxidative damage caused by stress conditions by increasing the antioxidative activity. The relationship between abiotic stress tolerance and an efficacious antioxidant system has been determined [26], however, the observance of the antioxidant system in salt-stressed plants treated with ACCD-producing bacteria has received too little attention. Therefore, in the present study, we tried to explore the antioxidative defense response of salt-stressed wheat plant following treatment with ACCD-producing bacterium.

Moreover, in response to abiotic stressors, plants defend themselves through the accumulation of certain osmolytes or compatible solutes. The accumulation of osmolytes helps plants for osmotic adjustment to cope with abiotic stressors [27]. Certain PGPR modulate the osmolytes to protect plants from salt stress. Zhang et al. [28] has demonstrated that soil bacterium *Bacillus subtilis* GB03 improved the osmotic stress tolerance in *Arabidopsis* by elevating the level of endogenous osmoprotectants. Similarly, ACCD-producing bacteria *Aneurinibacillus aneurinilyticus* and *Paenibacillus* sp. significantly reduced the salt stress stimulated ethylene levels and improved the growth of *Phaseolus vulgaris* plants [29]. The association of metal resistant microbe with ACCD activity is used as a tool for phytoremediation technology to enhance the metal tolerance and increase the yield in plants [30]. Previous reports [31, 32] suggested that a few bacterial strains such as *Serratia* sp., *Pseudomonas* sp., and *Bacillus* sp., modulate the mobilization of Zn in soybean and wheat plants. Islam et al. [33] showed that *P*. *aeruginosa* boost the stress tolerance in wheat plants under high Zn stress. Similarly, *Bacillus megaterium* and *Neorhizobium huautlense* T1–17 stimulated the growth of *Brassica juncea* and cabbage under Ni [34] and Pb stress respectively [35].

Among the staple crops, wheat production in India is approximately 80.2 million tonnes (http://www.agricoop.nic.in) which correspond to about 12% of total world production (http://dacnet.nic.in). Like many other crops, germination of wheat seed and seedling growth are severely affected by salt and metal stress worldwide [36]. The various conventional methods are in practice for alleviating salt stress, but most of them are costly and deleterious to environments. The micro-organisms residing in the rhizosphere have proved to regulate plant growth under normal and stress conditions. Therefore, the present study aimed to investigate the effectiveness of ACCD-producing bacterium *Enterobacter cloacae* ZNP-4 as a biological tool for alleviating the adverse effects of abiotic stressors and examined for its potential to alleviate stress-induced plant growth inhibition.

## Material and methods

### Isolation of ACC utilizing bacteria and enzymatic assay

ACCD-producing bacterium was isolated from the rhizosphere soil of *Ziziphus nullifera* growing in the desert of Rajasthan (28.13°N, 75.4°E) following the standard protocol [16]. Screening for ACC utilization was tested by growing the bacterial isolate on DF-agar (Dworkin & Foster) plate supplemented with 3 mM ACC (Sigma-Aldrich, USA) as a nitrogen source. For confirmation of ACC utilization as a nitrogen source, the bacterial isolate was sub-cultured several generations on DF-ACC plate. The DF medium supplemented with the inorganic nitrogen source served as a positive control. In addition, utilization of ACC was confirmed by ninhydrin-ACC reaction [37, 38]. During the primary screening for ACC utilization, we screened a total of twelve morphologically different bacterial isolates. However, one isolate ZNP-4 possessing resistance to salt and metal (Zn) stress was selected for PGP traits, biochemical characterization and its ability to protect wheat plant under salt and Zn stress.

### Taxonomical identification and phylogenetic analysis

Total g-DNA of ZNP-4 was extracted by standard DNA isolation kit (Qiagen, USA) following the manufacture protocol. The 1.5 Kb of 16S rRNA gene amplification was performed with primers, 27 F1 and 1494 Rc following standard procedure. The purified PCR product was sent to Xcelris Genomics Labs Ltd (Xcelris, India) for sequencing. Taxonomic affiliation of ZNP-4 was confirmed by the RDP database at a 98% threshold. Furthermore, phylogeny was established by software MEGA 6.0 [39].

### Screening for PGP traits

Spectrophotometric production of IAA was evaluated by Salkowsky’s reagent method at 530 nm using a Jasco-630 UV-visible spectrophotometer (Jasco Corporation, Japan) [40]. The phosphate solubilization ability of ZNP-4 was tested in a broth medium supplemented with in-organic phosphate [41]. The release of free phosphate was quantified as compared to a standard curve of K_2_HPO_4_ [42]. Siderophore production was tested by chrome azurol S agar (CAS) described by Schwyn and Neiland [43]. Primary screening to fix atmospheric nitrogen was evaluated by growing the bacterium on a semi-solid JNFb^-^/LGI medium at 28°C for 7 days [44]. Ammonia production ability of strain was determined by Nessler’s reagent method [45]. The screening for HCN (hydrogen cyanide) production was calculated by filter paper soaked with picric acid (0.5%) and Na_2_CO_3_ (2%) solution [46]. Antagonistic activity against bacterial and fungal pathogens was determined by the well diffusion method. Antagonistic activity against bacterial pathogens such as *Bacillus cereus*, *Erwinia carotovora*, *Escherichia coli*, and *Staphylococcus aureus* was determined by growing them on LB-agar plates at 37°C for 24 h. The boiled culture was used as a control and the experiment was performed in triplicate. To test the antifungal activity, 100 μl fungal spores of *Aspergillus flavus*, *Candida albicans*, *Colletotrichum caspasci*, *Fusarium oxysporum*, *Fusarium moniliforme*, *Fusarium graminearum*, and *Penicillium citrium* was suspended in sterile 0.85% saline solution and further spreaded on the potato dextrose agar (PDA, Himedia, India) plate. With the help of a metallic borer, wells of 6 mm diameter were made which were then filled with the overnight grown culture of the isolate (1×10^8^ CFU/ml) and kept for incubation at 28°C for seven days. Test organism was screened for various biochemical tests as per standard protocols [47]. Screening for sensitivity to various antibiotics and carbohydrate utilization efficacy was checked by respective kits (HTM 002, KB 009, Himedia). The motility of isolate ZNP-4 was tested using a standard protocol of Connelly et al. [48].

### Physiological characterization of ACCD activity

ACCD activity of isolate ZNP-4 was checked in diverse physiological conditions such as substrate ACC (1–5 mM), salt concentrations (2–8%), pH (3–12), the incubation period (0–72 h) and Zn stress (2–8%) following the standard protocol [16]. The culture medium pH was adjusted with 2 N HCl and 1 M NaOH to attain pH 5.0 to 10.0 using the pH meter (Eutech, pH 1100). ZnSO_4_ was used for imposing metal stress. The ACCD-production was evaluated by measuring the amount of α-ketobutyrate (KB) produced by enzymatic hydrolysis of ACC. The produced α-KB was determined at 540 nm to the standard curve of α-ketobutyrate (Sigma-Aldrich, USA) generated in the range of 0.1 to 1.0 μmol. Cultures of equal OD (OD_600_) were used for ACCD assay.

### Growth curve analysis

The isolate was screened for its ability to tolerate various abiotic stressors. Overnight grown culture (20 μl) of ZNP-4 was grown into DF medium with different concentrations of NaCl (2 to 8%). For temperature tolerance, ZNP-4 was grown in DF-medium and incubated at different temperatures (30°C to 40°C). Similarly, the test organism was grown in DF-medium with a wide pH range of 3–12. The growth analysis under Zn stress was evaluated by growing the isolate in SLP medium amended with different concentrations of Zn (2 to 10%). Cultures in each treatment were grown for 24 h and absorbance of the culture was determined at 600 nm in triplicate sets using un-inoculated broth as a blank.

### Plant growth promotion test under salt stress

#### Soil characteristic and plant growth test

For plant growth studies, the soil was analyzed for its various parameters including, organic carbon [49], phosphorus content [50] and availability of other nutrients such as nitrogen, potassium and micronutrients (Fe, Cu, Zn, and Mn) [51].

#### Plant inoculation

Based on various PGP traits, the test organism was used for its ability to enhance *Triticum aestivum* growth in a plant growth chamber (Labtech, South Korea). Inoculum preparation and seed treatment was carried out according to Penrose and Glick [16]. The soil was sterilized by autoclaving at 121°C for three times to kill the entire microorganism. Bacterized seeds of approximately twenty seeds were sown in plastic pots filled with sterile soil (400 g) in triplicates in a growth chamber with 16:8 photoperiods at 28±2°C. Hoagland medium supplemented with salt (150 mM; treatment T1 & 200 mM; treatment T2) was used for providing nutrient as well as imposing salt stress on each alternate day [52]. For Zn stress, the sterilized soil was mixed with ZnSO_4_ solution to attain the final concentration of 150 (treatment T1), and 250 mg kg^−1^ (treatment T2) and left for 10 days for metal stabilization. Pots were arranged in a completely randomized block design with three replicates in each treatment. The experiment was conducted for 15 days after the seed germination. Various physiological parameters and chlorophyll content were quantified [53].

#### Antioxidative enzyme assay

The enzymatic antioxidant of wheat plants was extracted by thoroughly grinding the leaves (0.5g) in 5 ml of cooled potassium phosphate buffer (50 mM, pH 7.8). The homogenate was centrifuged at 10,000g for 20 mins at 4°C to collect the supernatant. The reaction mixture (3 ml containing 50 mM phosphate buffer (pH 7.8), 13 mM methionine, 75 μM NBT, 2 μM riboflavin, 0.1 mM EDTA) and 100 μl of enzyme extract were used for detection of the SOD enzymatic assay [54]. A reaction mixture lacking the enzyme extract was used as a control. The reaction mixture was kept under a fluorescent lamp (30 W) for 10 min and the further reaction was stopped by turning off the light. One unit of enzyme activity was taken as the amount of enzyme that inhibited 50% of NBT photo-reduction at 560 nm.

For CAT activity, the reaction mixture (3 ml containing 50 mM phosphate buffer (pH 7.8), 0.1 mM EDTA, 12.5 mM H_2_O_2_) and 100 μl enzyme extract were mixed and a decrease in an absorbance was read at 240 nm. One unit of enzyme activity was taken as absorbance change of 0.01 min per min. Peroxidase (POD) activity in the reaction mixture (3 ml containing 0.1 M phosphate buffer, 0.1 mM pyrogallol, 5 mM H_2_O_2_) and 100 μl of enzyme extract was mixed and incubated for 5 min at 25°C. The reaction was stopped by adding 1.0 ml of 2.5 N H_2_SO_4_. The absorbance of indigo colour formed was read at 420 nm against blank.

#### Biochemical analysis of plant

For proline assay, leaf samples (0.5 g) were homogenized in 3% sulphosalycylic acid. Following filtration, ninhydrin (2%) and glacial acetic acid were added and it was heated at 100°C for 1 h in a water bath. Following extraction with toluene, the absorbance was read at 520 nm [55]. The proline content was compared to the standard curve generated using L-proline (Sigma-Aldrich, USA). For determination of lipid peroxidation, alcoholic extract of leaf samples (0.5 g) was mixed with 1 ml of 0.5% thio-barbituric acid containing 20% trichloroacetic acid and it was heated up to 90°C for 30 min. Following centrifugation at 5,000 g for 5 min, the absorbance was recorded at the wavelength of 400, 532 and 600 nm, respectively. MDA content was calculated by its molar extinction coefficient (155 nm ^-1^cm^-1^) and the results were expressed in terms of mmol MDA g^-1^ FW [56].

#### Measurement of hydrogen peroxide and superoxide

Leaf tissue (0.5g) was extracted in phosphate buffer (pH 6.5) and following centrifugation, supernatant (3 ml) was mixed with 1 ml of titanium sulphate sulfate (0.1% in 20% H_2_SO_4_). The absorbance of the mixture was recorded at 410 nm. The extinction coefficient (0.28 μM^-1^cm^-1^) was used to calculate the extent of H_2_O_2_ in terms of μmol g^-1^ fresh weight [57]. Similarly, 0.5g of leaf tissue in each treatment was grounded in liquid nitrogen. The grounded paste was suspended in 50 mM of phosphate-buffered saline (PBS) solution and following centrifugation, the obtained supernatant was used for O_2_^-^ measurement.

#### Test of colonization

Roots sections of bacterial inoculated wheat plants were cut into smaller segments and an appropriate amount (1 g) was dipped into sterilized PBS buffer (5 ml) and vortexed to release the bacteria into the buffer. Further, it was serially diluted and poured on a nutrient agar plate to evaluate the population of indigenous bacteria. The colony forming units (CFU) were counted after 24 to 48 h of incubation at 30±2°C.

### Statistical analysis

Data of the tested experiments were analyzed by analysis of variance (ANOVA) and Duncan’s multiple range test. The significance level of P = 0.05 was used for comparing the means for all analyses.

## Results

### Isolation and plant growth promoting features

Isolate ZNP-4 was selected based on its ability to grow on DF-ACC amended medium. ACCD activity of isolate was further confirmed by enzymatic assay, which showed the activity of 188.90±7.30 nmol of α-ketobutyrate (KB) mg^-1^Pr^.^ (protein) h^-1^. The appearance of halo-zone around the bacterial colony on NBRIP-agar medium containing tricalcium phosphate indicated phosphate solubilizing ability of ZNP-4. It was further quantified as 13.35±3.05 μg ml^-1^. The test organism showed the production of phytohormone indole-3-acetic acid (0.364±0.02 μg ml^-1^) (Table 1). However, it showed negative for siderophore production. The luxuriant growth on the JNFb^-^/or LGI medium was considered as positive for nitrogen fixation. The isolate was also found to be positive for ammonia and HCN production.

**Table 1:** Plant growth promoting traits of ZNP-4.

Plant growth promoting traits	Activity
ACCD activity (nmol of α-KB mg^-1^Pr^.^ h^-1^)	188.90±7.30
IAA production (μg ml^-1^)	0.364±0.02
Phosphate solubilization (μg ml^-1^)	13.35±3.05
Siderophore index	-
Chitinase activity	+
HCN production	+
Ammonia production	+

### Identification and phylogenetic analysis

**Fig 1:**
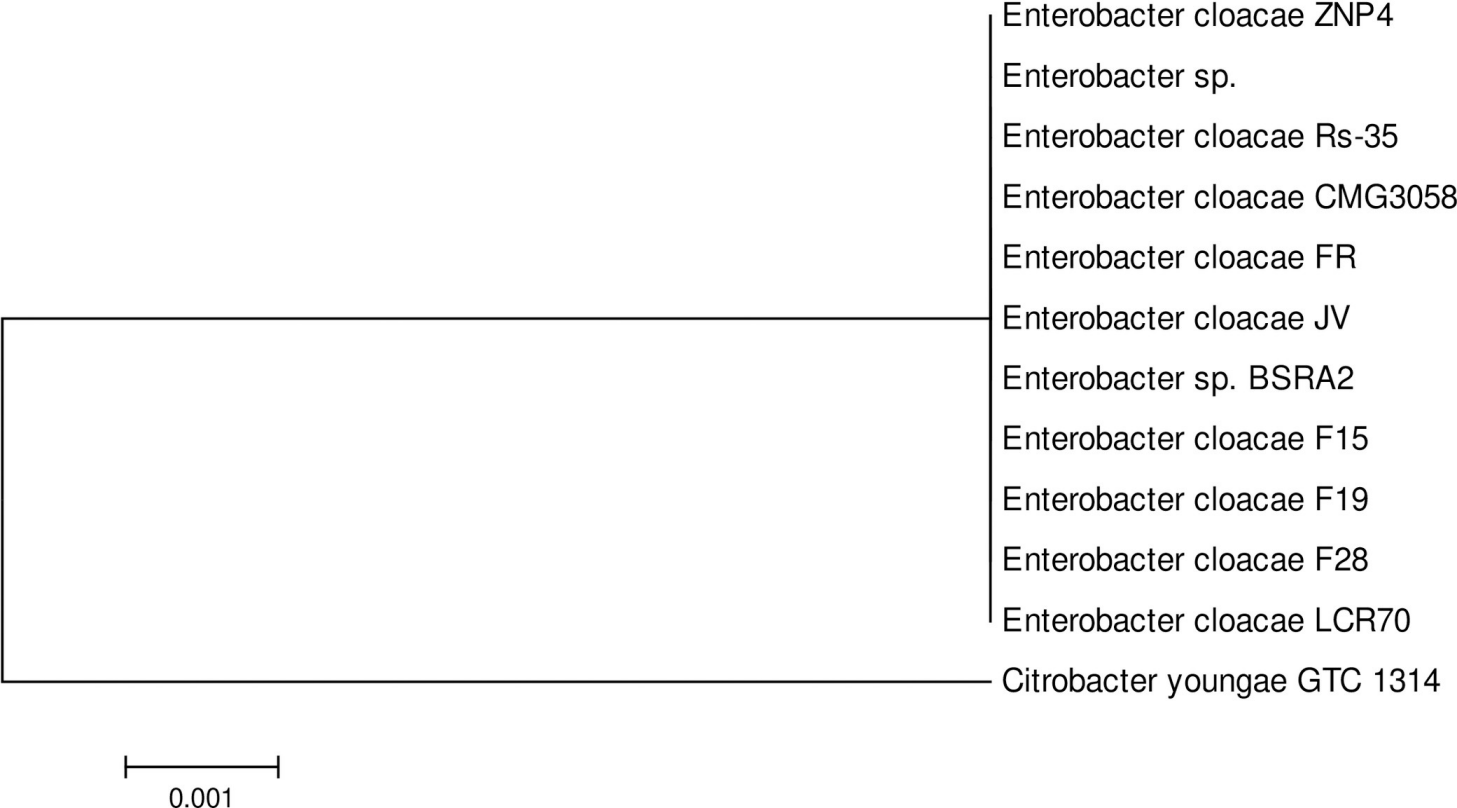
Phylogenetic tree of *Enterobacter cloacae* ZNP-4 using 16S rRNA gene sequences. The tree was constructed by neighbor-joining method at bootstrap value of (n = 500) using the software packages Mega version 6.0.

Based on 16S rRNA gene sequencing, the isolate was identified as *Enterobacter cloacae* which showed 99% similarity with the 16S rRNA gene sequence of type strains *Enterobacter cloacae* RS-55 and CMG3058. Based on phylogenetic analysis, the strain was showing closest relative to other *Enterobacter cloacae* and *Enterobacter* sp. (Fig 1). The sequenced 16S rRNA nucleotides have been deposited in the GenBank database with accession number KJ950705.

### Physiological pattern of ACCD activity

**Fig 2:**
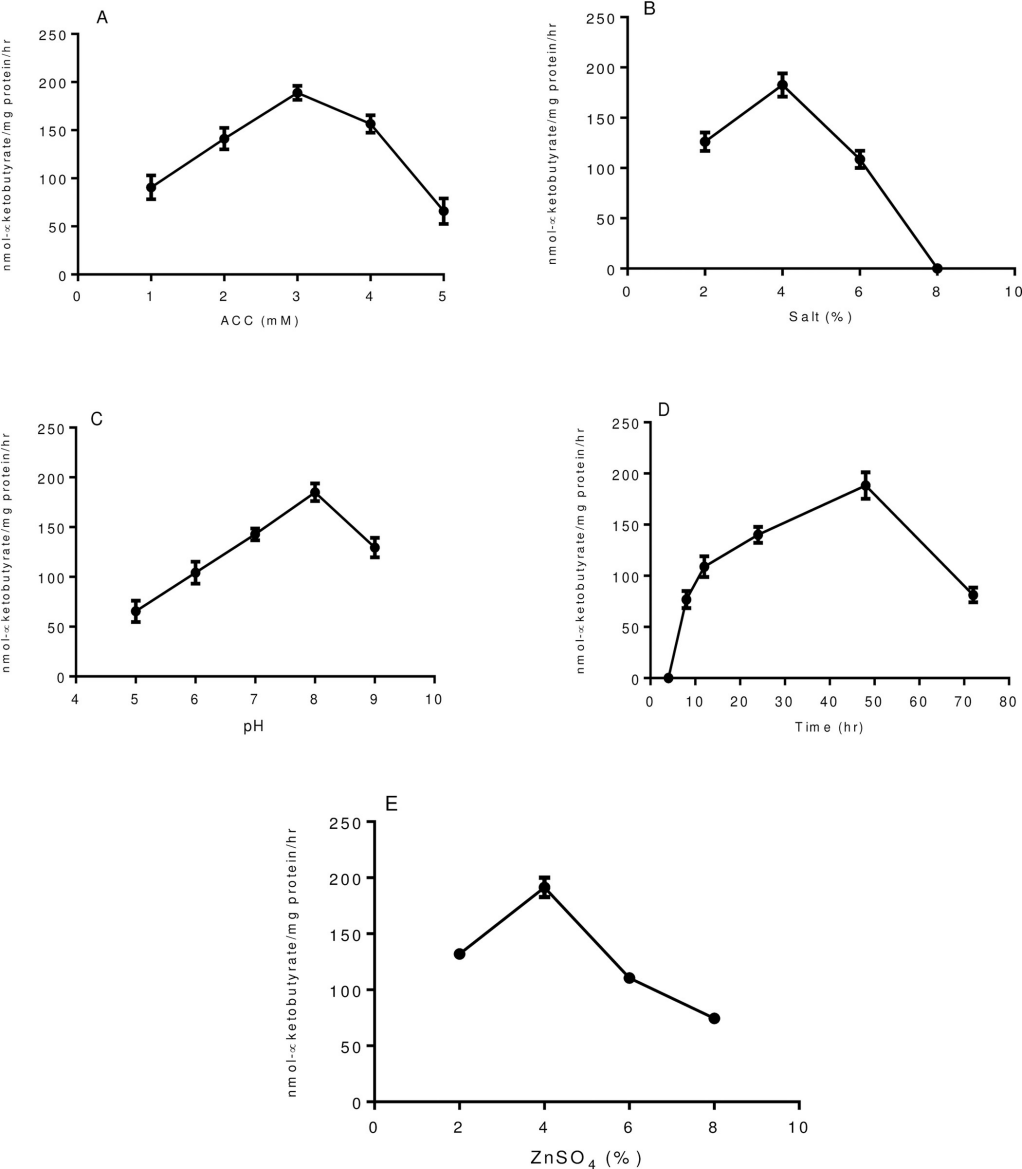
Pattern of ACCD activity of isolate ZNP-4 under varying physiological conditions (A) substrate (ACC) concentration (B) at different salt (NaCl) concentrations (C) varying pH conditions (D) under different time period (E) different concentration of ZnSO_4_. Each value represent the mean of three replicates±SD (n = 3).

ACCD activity was tested under different substrate conditions and it was found to be optimum (188.90±7.30 nmol of α-ketobutyrate mg^-1^Pr^.^h^-1^) at 3 mM ACC concentration (Fig 2A). A decrease in ACCD activity (187%) was observed with an increase in ACC concentration of 3 mM to 5 mM. ACCD activity was evaluated at different salt concentrations, which showed an increase in salt concentration from 2% to 4%, enzymatic activity increased upto 45%, however, a decrease in ACCD activity of 68% was observed with an increase in NaCl concentration 4% to 6% (Fig 2B). Optimum enzyme activity (184.95±8.80 nmol of α-ketobutyrate mg^-1^Pr^.^h^-1^) was recorded at pH (8.0) (Fig 2C). ACCD activity was evaluated in the different incubation periods and the highest activity (188.29±12.9 nmol of α-ketobutyrate mg^-1^Pr^.^h^-1^) was obtained after 48 h of the incubation period. Further incubation at 72 h time of period, enzymatic activity decreased upto 132% (Fig 2D). The highest enzyme activity was recorded at 4% ZnSO_4_ concentration, however a decrease (70–150%) in ACCD activity was observed with an increase in ZnSO_4_ up to 8% (Fig 2E).

### Biochemical analysis

**Fig 3:**
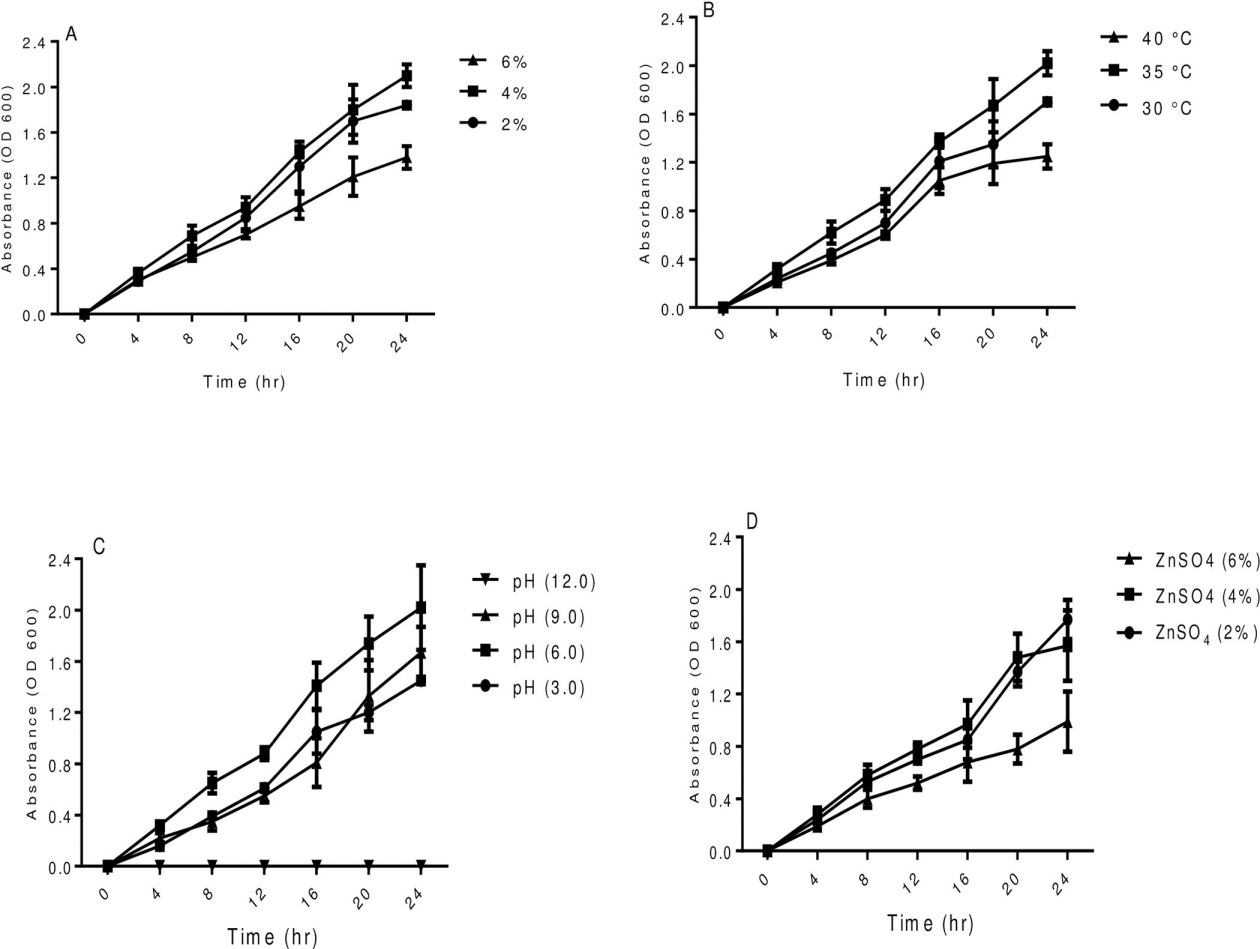
Growth curve of ZNP-4 under different conditions (A) varying concentration of NaCl (B) different temperature (C) different pH (D) ZnSO_4_ concentrations. Each value represent the mean of three replicates±SD (n = 3).

The test isolate ZNP-4 was found to be gram-negative and showed positive for various tested biochemical parameters, which has been summarized in Table 2. Similarly, carbohydrate utilization ability and antibiotic profiling of the tested strain has been determined (Table 2). The growth analysis suggested that isolate can grow up to 6% of salt stress, while the optimum growth was noticed at 4% NaCl (Fig 3A). We could not observe growth at 8% NaCl stress. Among the temperature treatments, the optimum growth was recorded at 35°C (Fig 3B), however the test isolate did not show growth at 45°C. Similarly, at different pH, optimum growth was observed at pH 6.0. Further increase in pH 6.0 to 9.0, decrease in growth was observed (Fig 3C). The growth pattern of test isolate at different ZnSO_4_ showed optimum growth at 2 and 4%, however a decrease in growth was observed with a further increase in ZnSO_4_ (6%) (Fig 3D). The test organism showed antifungal activity against *Aspergillus flavus*, *Fusarium oxysporum*, *Fusarium moniliforme*, *and Penicillium citrinum*. However, it did not show antibacterial activity against the tested pathogenic bacterial strains (S1 Table).

**Table 2:** Biochemical characteristic feature of ZNP-4.

Characteristic (s)	Activity	Carbohydrate	Activity
Gram reaction	-	Sodium gluconate	-
Catalase	+	Glycerol	+
Indole	-	Salicin	+
MR	-	Dulcitol	-
VP	+	Inositol	+
Amylase	-	Sorbitol	+
Lipase	-	Mannitol	+
Urease	+	Adonitol	+
Nitrate reductase	+	Inulin	+
Oxidase	-	Arabitol	-
Temp. tolerance (°C)	45	Erythritol	-
Salt tolerance (%)	6%	Citrate	+
pH	4–10	α-Methyl-D-glucoside	-
Swimming	+	L-Arabinose	+
Swarming	+	Rhamnose	+
Twiching	+	Cellobiose	+
**Carbohydrate**	**Activity**	Melezitose	+
Lactose	+	α-Methyl-D-mannoside	-
Xylose	+	Xylitol	-
Maltose	+	ONPG	-
Fructose	+	Esculin hydrolysis	+
Dextrose	+	Mannose	+
Sucrose	+	D-Arabinose	-
Galactose	+	Malonate utilization	+
Raffinose	-	Trehalose	+

### Plant growth promotion test under salt and metal stress

**Fig 4:**
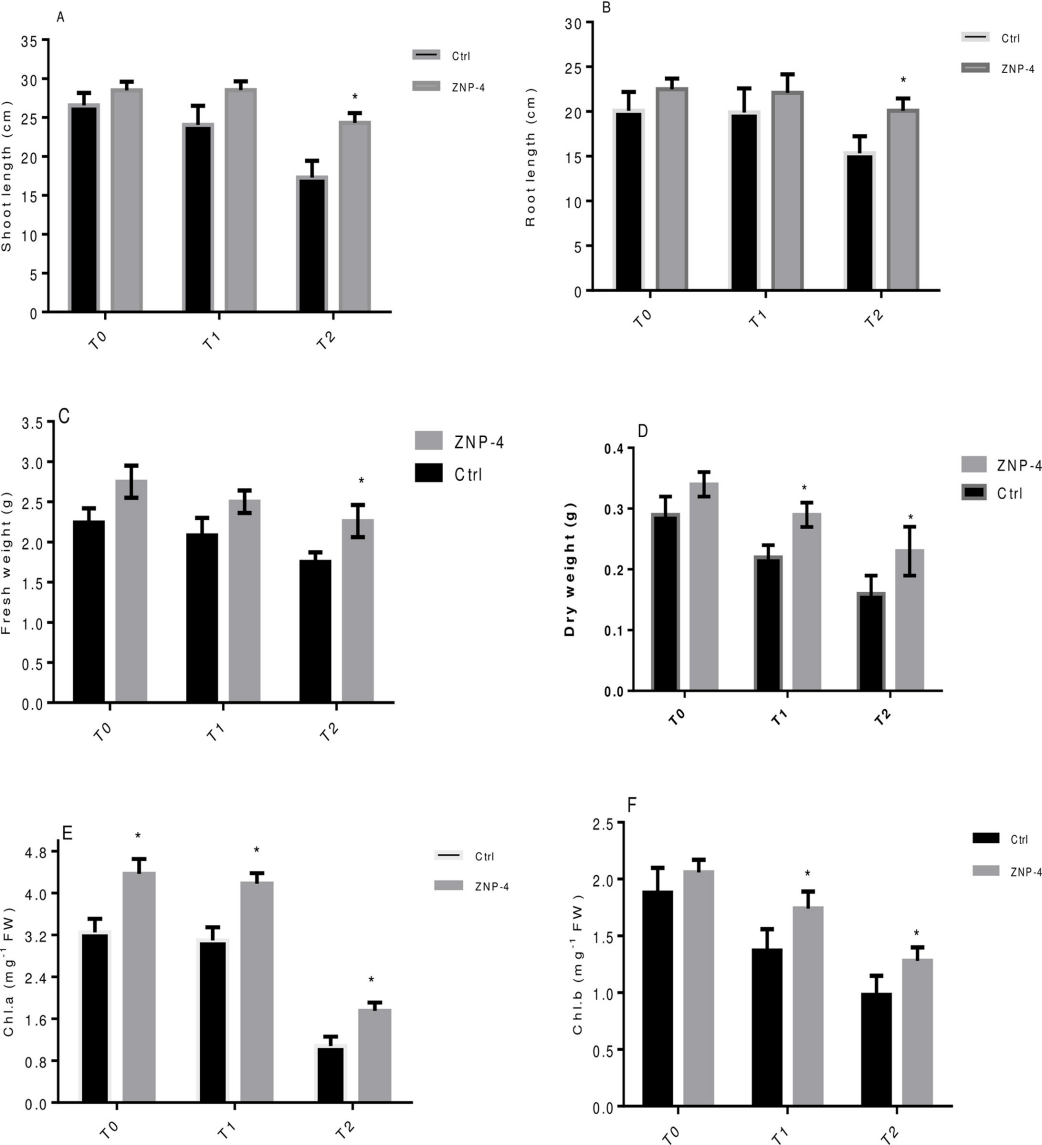
Effect of inoculation with bacterium ZNP-4 on plant growth and chlorophyll contents under different salinity conditions (T0: 0 mM; T1: 150 mM, T2: 200 mM NaCl) (A) shoot length (B) root length (C) fresh weight (D) dry weight (E) chlorophyll a (F) chlorophyll b. Values are mean±SD of triplicate sets (n = 15). * represent the significant difference as compared to respective control according to Ducan’s multiple range test (p = 0.05).

Bacterium inoculation significantly improved the growth of wheat plants under the various levels of salinity stress. Physiochemical properties of soils used for pot experiment have been summarized in S2 Table. Considering the shoot length, the highest significant (p = 0.05) increase in growth of 40.65% was observed at treatment T2 in bacterium inoculated plants as compared to respective un-inoculated control (Fig 4A). Similarly, in the case of root length, a significant (p = 0.05) increase in root length (30.96%) was observed at treatment T2 in bacterium treated plants as compared to their respective control (Fig 4B). ZNP-4 inoculation significantly (p = 0.05) improved the fresh weight with highest increase of 28.30% was observed at 200 mM NaCl (treatment T2) (Fig 4C). Inoculation with isolate ZNP-4 significantly improved the dry weight content of 43% (p = 0.05) and 32% (p = 0.05) at treatment T2 and T1 (Fig 4D). The effect of ZNP-4 inoculation was also evaluated on the photosynthetic pigments particularly chlorophyll a and b. Highest significant (p = 0.05) increase in chlorophyll a (62.03%) was observed at 200 mM (treatment T2), followed by 34.83% at 150 mM NaCl (treatment T1) stress as compared to respective control (Fig 4E). Increase in chlorophyll a content was 34.46% (p = 0.05) at 0 mM salt stress in inoculated plants. Significant (p = 0.05) increase in chlorophyll b was 26.00% at 150 mM NaCl (treatment T1) stress as compared to respective control plants (Fig 4F).

**Fig 5:**
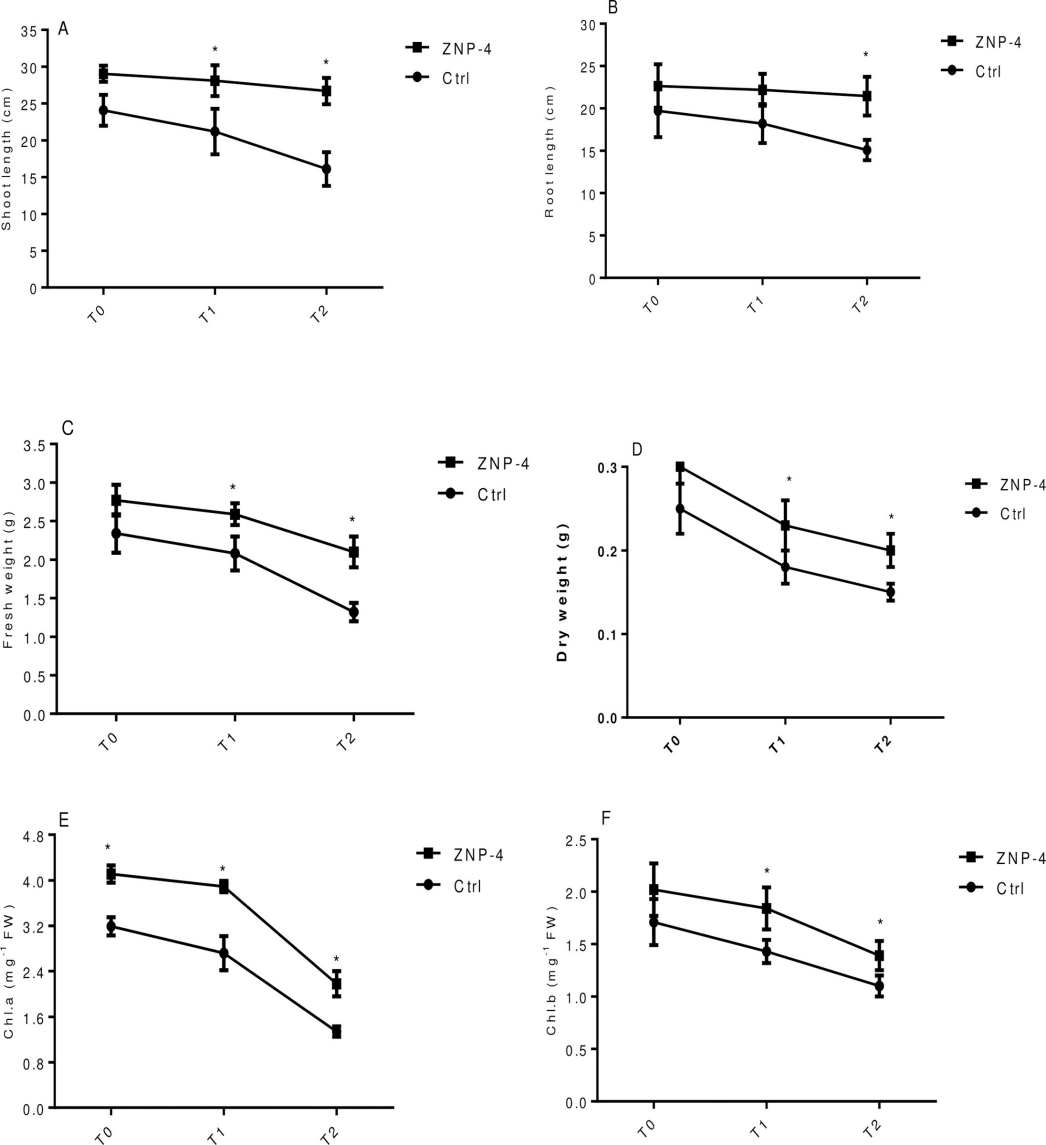
Effect of inoculation with bacterium ZNP-4 on plant growth and chlorophyll contents under different metal (ZnSO_4_) concentrations, (A) shoot length (B) root length (C) fresh weight (D) dry weight (E) chlorophyll a (F) chlorophyll b. Values are mean±SD of triplicate sets (n = 15). * represent the significant difference as compared to respective control according to Ducan’s multiple range test (p = 0.05).

Under Zn stress, the highest significant (p = 0.05) increase in shoot length of 65% was recorded at treatment T2, followed by 32% at treatment T1 in bacterial inoculated plants as compared to respective control (Fig 5A). Bacterial inoculation significantly (p = 0.05) increased the root length (45%) at treatment T2 (Fig 5B). Increased in fresh weight was 54% (p = 0.05) and 24% (p = 0.05) at treatment T2 and treatment T1, respectively (Fig 5C). A significant increase in dry weight was recorded of 33% and 27.7% at treatment T2 and T1 under metal stress (Fig 5D). Similarly, a significant (p = 0.05) increase in chlorophyll a was 28%, 43% and 62% at T0, T1 and T2 treatments in bacterial inoculated plants, respectively (Fig 5E). ZNP-4 inoculation significantly increased the chlorophyll b (29%) at treatment T1, followed by 26% at treatment T2 (Fig 5F).

### Antioxidant activities under salt and metal stress

**Fig 6:**
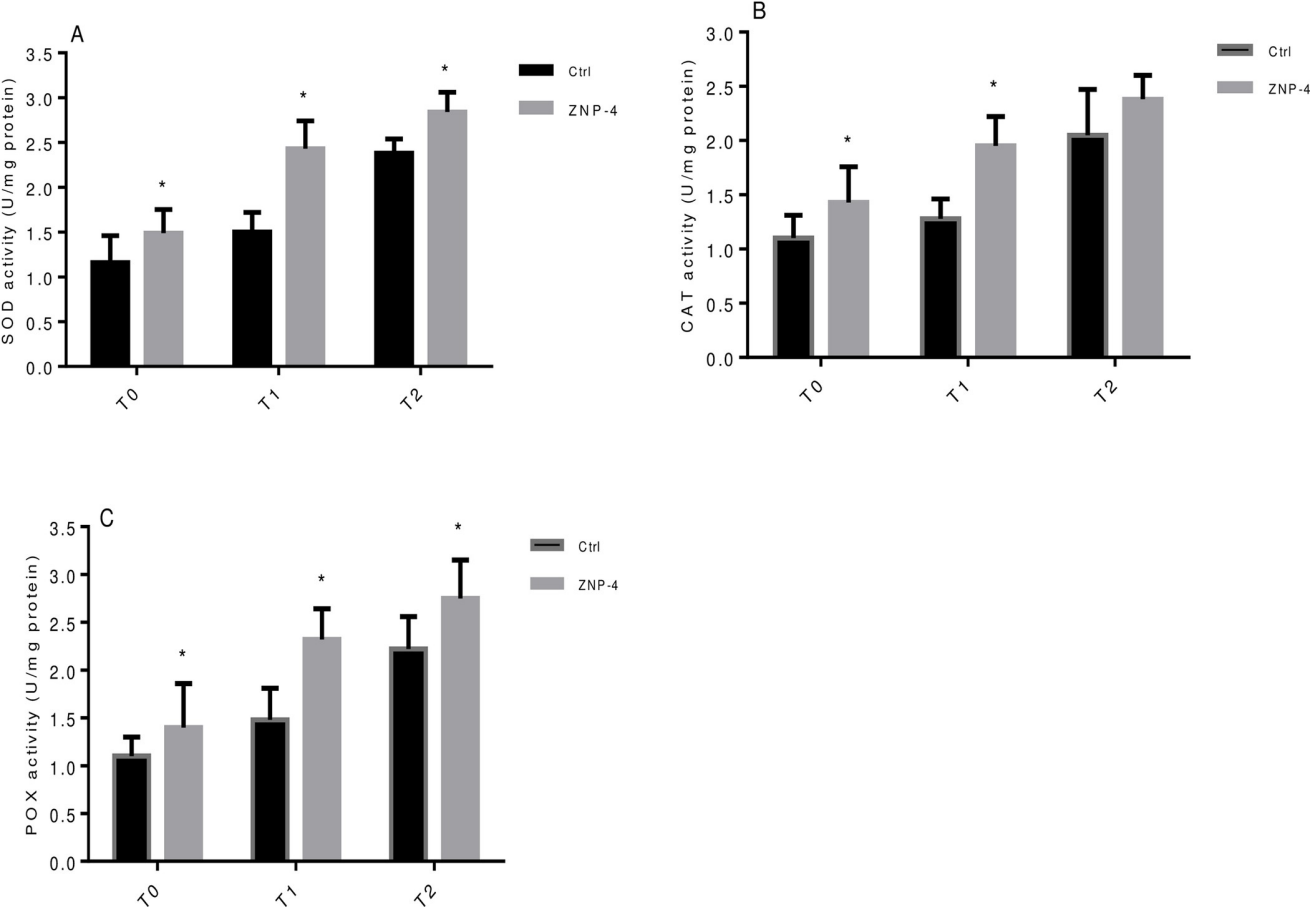
Effect of bacterium inoculation on antioxidative activities under tested salinity stress conditions (A) super-oxide dismutase (B) catalase (C) peroxidase. Each value represent the mean±SD of triplicate sets (n = 15). The significant difference compared to corresponding control has been denoted by *. Error bar represent the standard deviation of triplicate sets with five measurement in each set (n = 15).

Significant increase in the anti-oxidative activity of SOD, CAT and POX was observed following inoculation of ZNP-4 under tested salinity conditions. As seen from Fig 4A, ZNP-4 inoculation significantly (p = 0.05) increased the SOD activity of 28.7%, 57.2%, and 20.8% at treatment T0, T1 and T2 as compared to respective control plants, respectively (Fig 6A). Similarly, significant increase (p = 0.05) in catalase activity was recorded 33% and 44.5%, at treatment T0 and T1, respectively as compared to control plants treated with respective salt stress (Fig 6B). Increase (p = 0.05) in POX activity of 29.7%, 42.2%, and 21.9% was recorded at treatment T0, T1 and T2, respectively (Fig 6C).

**Fig 7:**
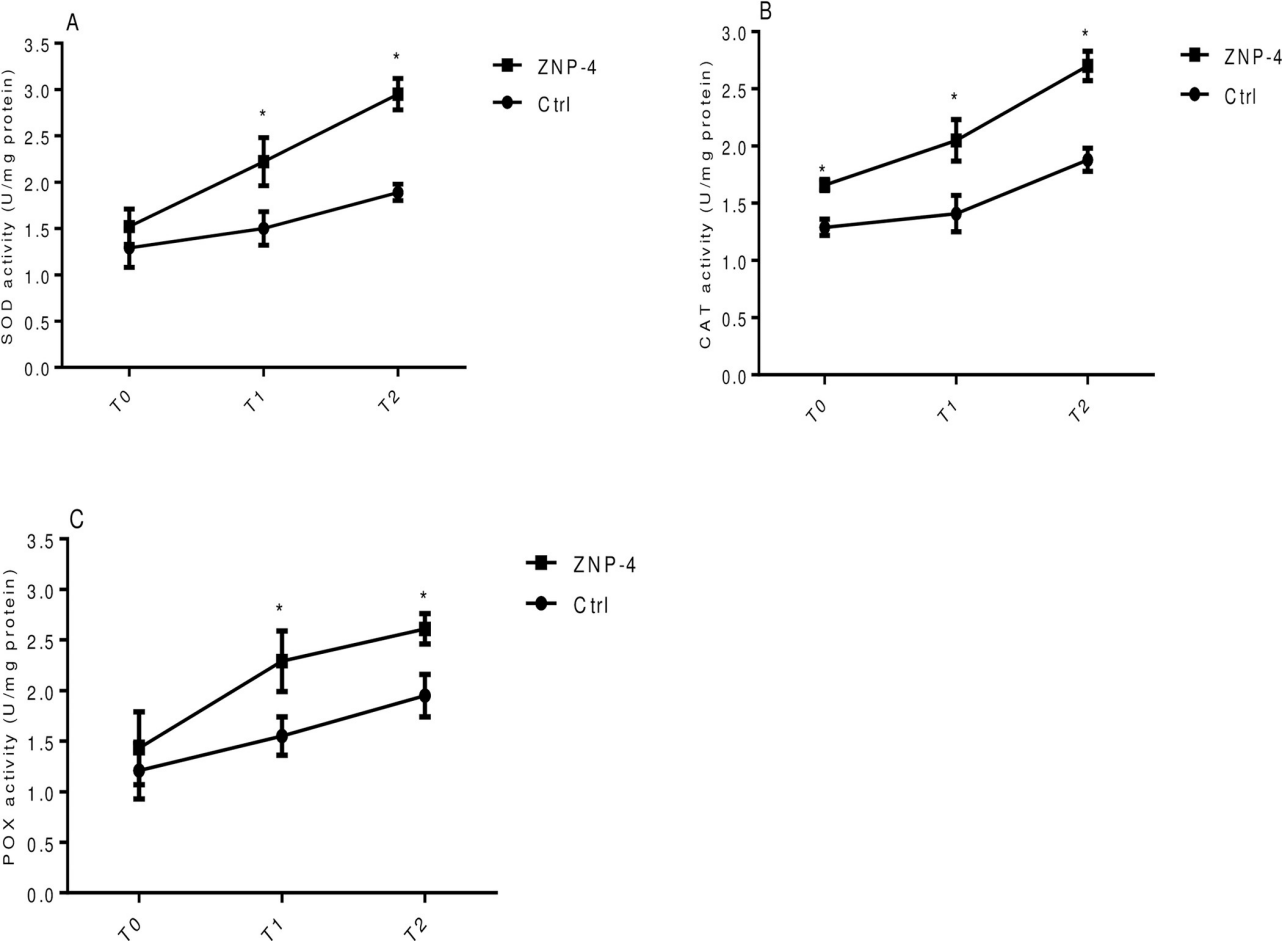
Effect of bacterium inoculation on antioxidative activities under tested metal stress (A) super-oxide dismutase (B) catalase (C) peroxidase. Each value represent the mean±SD of triplicate sets (n = 15). The significant difference compared to corresponding control has been denoted by *. Error bar represent the standard deviation of triplicate sets with five measurement in each set (n = 15).

Under metal stress, bacterial inoculation significantly (p = 0.05) increased the SOD activity of 48% and 56% at treatment T1 and T2, respectively (Fig 7A). Increase in CAT activity was 28%, 45% and 43% at treatment T0, T1 and T2, respectively (Fig 7B). Bacterial inoculation also increased the POX activity at treatment T1 (47%) followed by treatment T2 (34%) (Fig 7C).

### Biochemical analysis under salt and metal stress

**Fig 8:**
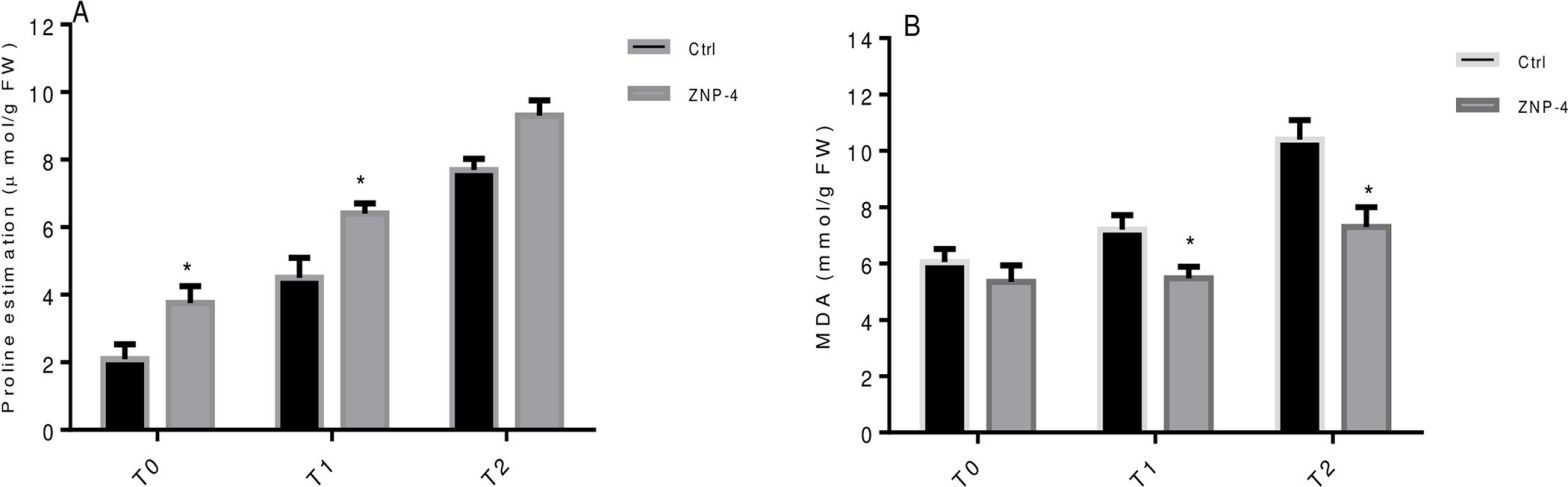
Effect of bacterium inoculation on proline and malondialdehyde content under tested salinity treatments (A) proline content (B) malondialdehyde content. Error bar represents standard deviation of five measurements in triplicate sets (n = 15). * represent the significant difference as compared to corresponding control as per Ducan’s multiple range test (p = 0.05).

**Fig 9:**
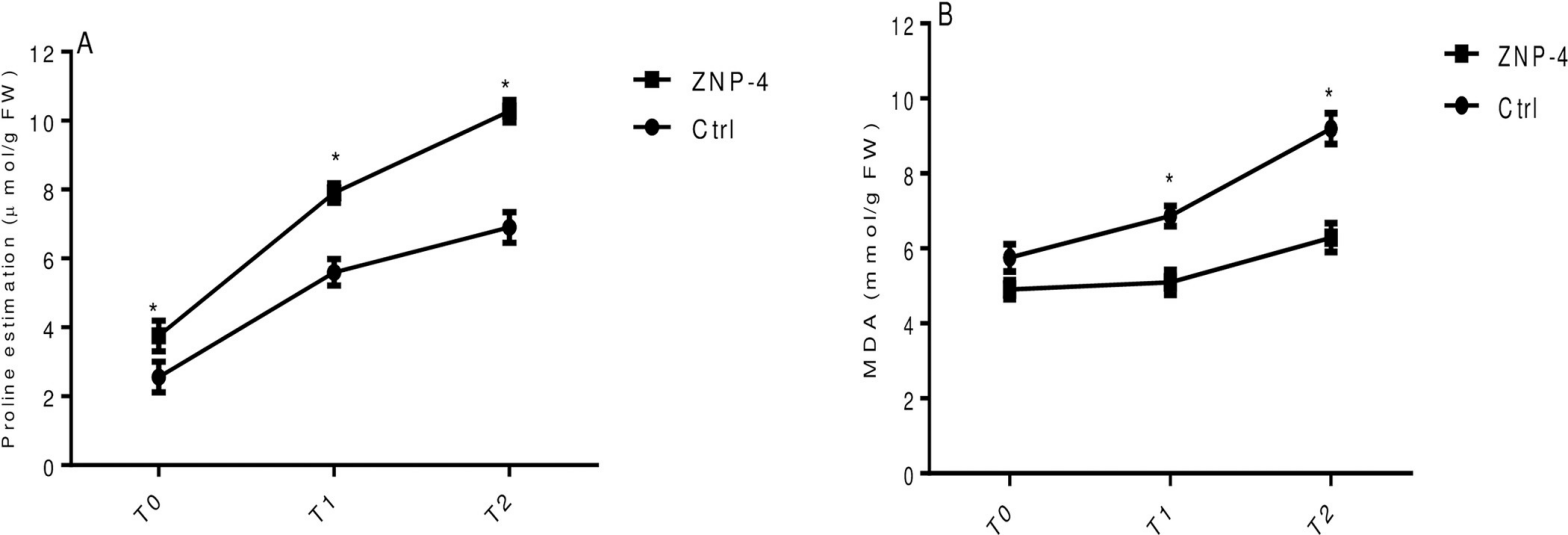
Effect of bacterium inoculation on proline and malondialdehyde content under metal stress (A) proline content (B) malondialdehyde content. Error bar represents standard deviation of five measurements in triplicate sets (n = 15). * represent the significant difference as compared to corresponding control as per Ducan’s multiple range test (p = 0.05).

Bacterium inoculation significantly increased the proline content in wheat plants under tested salinity stress. Highest significant (p = 0.05) increase of 31.5% and 22.7% was recorded at treatment T1 and T0 in bacterium inoculated plants as compared to their respective control (Fig 8A). Salinity also increased the MDA content of about 80.87% in salt-treated plants. However, ZNP-4 inoculation significantly decreased the MDA content of 45.50% and 35.28% (p = 0.05) at treatment T2 and T1 (Fig 8B). Similarly, effect of bacterial inoculation on proline and MDA content under metal stress was also tested. It is evident from Fig 9A that ZNP-4 inoculation significantly (p = 0.05) increased the proline content of 48.8% and 41.07% at treatment T2 and treatment T1, respectively. A significant increase in proline content of 33% was also observed at treatment T0. Considering the MDA content, the highest decrease of 46.4% and 35% was recorded at treatment T2 and T1, respectively, in tested bacterial inoculated plants (Fig 9B).

### H_2_O_2_ and O_2_^-^ content

**Fig 10:**
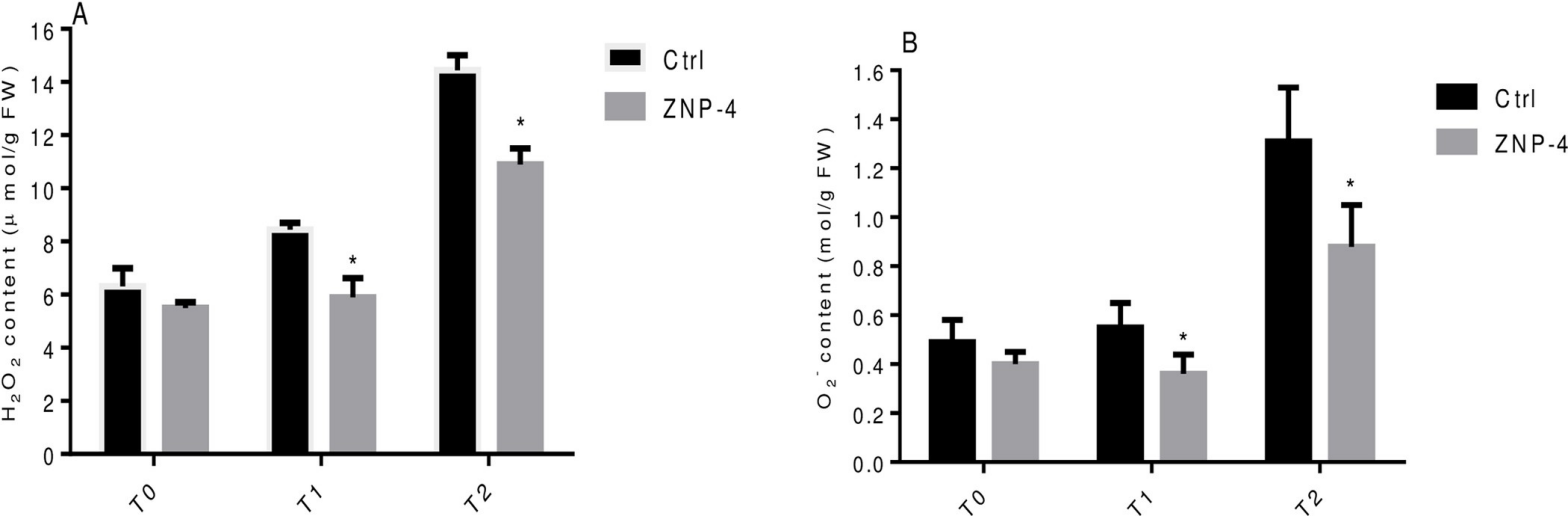
Effect of bacterium inoculation on H_2_O_2_ and O_2_^-^ level under tested salinity stress (A) H_2_O_2_ (B) O_2_^-^ content. * represent the significant difference as compared to respective control as per Ducan’s multiple range test (p = 0.05).

**Fig 11:**
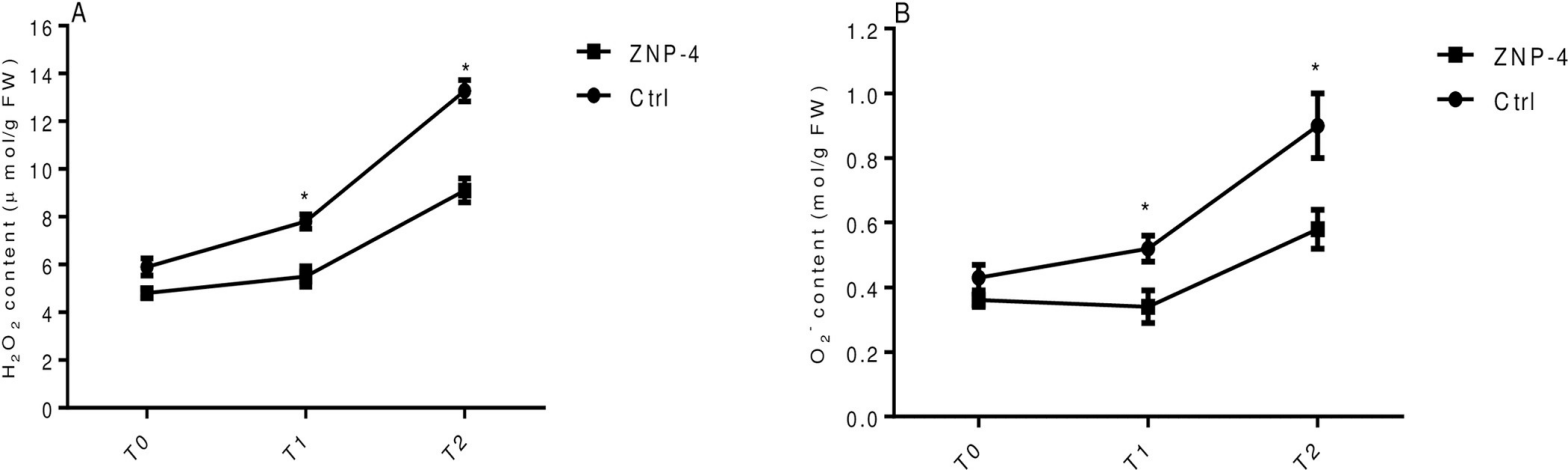
Effect of bacterium inoculation on H_2_O_2_ and O_2_^-^ level under metal stress (A) H_2_O_2_ (B) O_2_^-^ content. * represent the significant difference as compared to respective control as per Ducan’s multiple range test (p = 0.05).

Effect of bacterial inoculation on generation of ROS was monitored under salinity and metal stress treatments. The bacterial inoculation significantly reduced the H_2_O_2_ level under tested salinity stress. The highest significant (p = 0.05) decrease of 43.2% was recorded at treatment T1 followed by 32.5% at treatment T2. The reduction of H_2_O_2_ content was 14.7% at treatment T0 (Fig 10A). The salinity induced generation of O_2_^-^ content was also minimized with 52.7% (p = 0.05) and 49% (p = 0.05) at treatment T1 and T2 in bacterial treated plants (Fig 10B). Similarly, the decrease of oxidative stress in terms of reduced H_2_O_2_ and O_2_^-^ content under metal stress was observed following bacterial inoculation. The reduced content of 46% (p = 0.05) and 41.8% (p = 0.05) was recorded at treatment T2 and T1, respectively (Fig 11A). ZNP-4 inoculation reduced the oxidative damage by decreasing the O_2_^-^ content of 55% and 53% at treatment T2 and T1, respectively (Fig 11B).

### Colonization test

The ability of test isolate to colonize the wheat plant was determined by plating the suitable serial dilution of grounded wheat plant on LB-agar plate. After the experimental period, the associative bacteria were detected in range of 2.3×10^3^ CFU g^-1^ of root.

## Discussion

The use of PGPR offers an eco-friendly and valuable alternative to artificial fertilizers for ameliorating abiotic stress in plants. Being indigenous, and competent in the rhizosphere, these PGPR offers beneficial effects to the host plant via interaction and metabolism. One of the proposed mechanism through which PGPR enhances plant growth is the production of ACCD [21]. PGPR with ACCD activity has been reported to facilitate plant growth by reducing the stress-induced ethylene level and therefore enhancing plant tolerance to unwanted stress [21]. The test isolate ZNP-4 exhibited ACCD activity under diverse physiological conditions and therefore supported plant growth under stress conditions.

Plants growing under stress conditions face high nutrients and energy demands, and therefore, they invest more in metabolic processes to fulfil the additional demands. The isolate ZNP-4 was capable to produce IAA and solubilize in-organic phosphate that might mitigate the negative effects of salinity and help plants to cope with the additional nutrient demands. The ability to produce IAA has a profound effect on plant growth and development, including the promotion of root formation and proliferation to improve the water and nutrient uptake. The bacterial synthesized IAA along with endogenous IAA enhance the secretion of root exudates which serve as the energy source of root-associated bacteria and also improve their growth and colonization efficiency. The supplementary IAA reverses the growth inhibiting effects of various stresses as well as improves the photosynthetic performance. Phosphate solubilization in the rhizosphere is a valuable mechanism whereby PGPR increases nutrient availability to the host plants. The findings of the current study are in agreement with the previous report, where bacterial phosphate solubilization enhances plant growth [15]. A previous study suggested that both ACCD and IAA-synthetic genes are regulated by the sigma factor so that stress conditions might lead to an increase in their synthesis, which ultimately support plant growth under various stress conditions [38]. Additionally, strain ZNP-4 exhibited multiple PGP traits, which may promote plant growth in a promising way.

Our results of a significant increase in various plant growth parameters are in congruence with the previous report, where salt tolerance in plants was induced by PGPR [58]. Inoculation with ACCD-producing bacterium *P*. *putida* UW4 has been shown to enhance in growth parameters of canola plants under the inhibitory level of salinity stress [20]. The growth of plants is dependent on the availability of nutrients in the soils. The deficiency of nutrients resulted in ethylene generation in various plant tissues, however, even under nutrient depletion, PGPR act as an effective tool to promote plant growth [59].

PGPR can also indirectly support plant growth by protecting plants against various phytopathogens. Strain ZNP-4 showed antifungal activity against the tested fungal genera *Aspergillus*, *Fusarium* and *Penicillium* and thereby enhanced the development of defense response as well as induced systemic resistance. The test organism was found to be positive for catalase, which has the potential to alleviate the oxidative damage in roots caused by environmental stressors. The obtained results are particularly significant for the development of effective bio-inoculants for wheat growth.

The optimal enzyme activity was observed at different parameters such as 3 mM ACC concentration, pH 8.0 and 48 h of the incubation period. The observed results tallied with previous reports of Jha et al. [17] who evaluated ACCD activity in *Enterobacter* sp. The increase in growth and enzyme activity with increasing salt concentration would be beneficial to minimize the salinity-induced damages. Similar to our results, Tittaburt et al. [60] reported the enhanced *AcdS* gene expression following an increase in salinity. The findings indicate that regulation of *AcdS* gene might be under the control of the sigma factor by initiating expression of certain genes under stress condition [38].

Salinity reduces the plant’s ability to take up water and therefore leads to physiological drought conditions. Decrease in water availability results in a reduction in plant growth and photosynthetic rate. Salinity inhibits the growth and productivity of many plant species by severely affecting their physiological processes [61]. The photosynthetic performance of many plant species is severely damaged by salinity [62]. Under salinity stress, reduced photosynthesis is correlated with decreased chlorophyll contents and distortion of chlorophyll structures. In response to salt stress, a reduction in PS II activity has been reported in wheat plants [63]. In the present study, bacterium inoculation showed a significance increase in growth from 10% to 62% for different parameters tested. PGPR *Pseudomonas putida* UW4 has been shown to enhance the growth of canola plants grown under the inhibitory level of salt stressors [20]. Similarly, growth enhancement of *Limonium sinense* was also recorded following inoculation of ACCD-producing bacteria under salt stress [64]. The effectiveness of ACCD-producing bacteria to improve plant growth under metal stress has been carried out in many studies [65, 66].

The antioxidant enzymes SOD, CAT and POX are involved in the dis-mutation of H_2_O_2_ to water and oxygen, and therefore have a cumulative effect on the scavenging of abiotic stress-induced ROS generations [67]. The induction of antioxidants CAT and POX can be considered as one of mechanisms of stress tolerance in plants and play important roles in scavenging toxic ROS molecules generated by heavy metal and salt stress. SOD and POX are major antioxidant enzymes that resist lipid peroxidation and maintain membrane integrity. The previous report suggested that the activity of antioxidant enzymes including SOD and POX is positively correlated with stress tolerance [67]. In the present study, bacterial inoculation significantly increased the activity of antioxidant enzymes under gradient salinity and metal stress. The increased antioxidative activities at moderate salinity stress could quench the salinity induced H_2_O_2_ and protect the wheat plants from oxidative injury, while at higher salinity stress the scavenging functions of these enzymes were impaired.

Accumulation of compatible solutes or osmolytes under stress conditions enables plants to maintain proper osmotic balance [68]. In the present study, inoculation of ZNP-4 significantly increased proline content, indicating a higher degree of stress tolerance and protection host plants from stress-induced oxidative stress and cell damages. Proline in response to salinity stress acts as a ROS scavenger and osmotic stress regulator through the contribution of cellular osmotic adjustment, detoxification of ROS, stabilizations of proteins etc. [69]. Higher accumulations of proline content and enhanced antioxidative activities are associated with higher salinity tolerance in many plant species [70]. The increased accumulation of proline contents following ZNP-4 inoculation improved plant growth against salinity and metal stress.

Under stress conditions, the decomposition of polyunsaturated fatty acids of bio-membranes is reflected as enhanced production of MDA content. MDA is a cytosolic by-product of lipid peroxidation and its increased level act as a marker of stress-induced ROS production. MDA is normally used to assess the oxidative damages and degree of plant sensitivity to ROS-induced damages [71]. In the present study, enhanced peroxidation was observed in plants under metal and salinity stress, however, ZNP-4 inoculation decreased the MDA content, indicating lower membrane damages or higher stress tolerance of plants are induced by bacterial inoculation.

In response to salinity stress, the level of stress proteins either increases or decreases depending on plant species. Bacterial inoculation decreased H_2_O_2_ and O_2_^-^ content in wheat plant and maintained its level below the deleterious effect. At low concentration, H_2_O_2_ behave as a signalling molecule to modulate various genes involved in stress defense. It induces the mitogen-activated protein (MAP) kinase cascade which activates the antioxidant defense mechanism [72]. The enhanced antioxidant defense system could help in decreasing the endogenous contents of ROS including H_2_O_2_ and O_2_^-^ content, therefore minimizing the oxidative toxic effect [73, 74]. Our results provide an insight into plant growth promotion by reducing salinity and metal-induced growth inhibition through ACCD activity, thus broadening the range of functionality of plant-bacterial interactions.

## Conclusion

The observed results in the present study demonstrated that ACCD-producing bacterium ZNP-4 protects wheat plants against salt and metal stress. In response to test isolate inoculation, plants experience a multitude of changes and they often developed mechanisms to counteract salinity stress and establish the cross-tolerance. The inoculation of isolate ZNP-4 results in the synthesis of osmo-protectants, and eventually leads to restoration of cellular homeostasis. ZNP-4 inoculation enhanced the anti-oxidative activities and decreased the lipid peroxidation, indicating that these mechanisms adopt by wheat plants to counteract the stress effect are sufficient to prevent abiotic stress-induced oxidative damage. Additionally, the antagonistic potential of the isolate makes it a good candidate as a growth promoting agent for plants growing in desert environments. In conclusion, the present study provides compelling evidence that inoculation of wheat plants with *E*. *cloaceae* ZNP-4 significantly mitigated the imposed abiotic stress, thus confering induced systemic tolerance. Therefore, the use of such bacterium with multifarious PGP features could be useful to combat various stressors and hold a great promise to use as biofertilizers for metal contaminated soils. However, future work is required to evaluate the efficiency of isolate under actual field condition and also to test for further use in any specific applications.

## Supporting information

S1 Table Test of antagonistic activities against bacterial and fungal pathogens. (DOCX)

S2 Table Physiochemical properties of soil used for plant growth study. (DOCX)
