# Supplementary material for: ACC deaminase producing rhizobacterium Enterobacter cloacae ZNP-4 enhance abiotic stress tolerance in wheat plant
Source: PLoS One. 2022 May 6;17(5):e0267127. doi: 10.1371/journal.pone.0267127 (PMC9075627; doi:10.1371/journal.pone.0267127)
Supplement: S1 Table — (DOCX) [file pone.0267127.s001.docx]

**S1 Table**

**Test of antagonistic activities against bacterial and fungal pathogens**

**Bacteria Activity Zone of inhibition (mm)**

*Escherichia coli* -- NA

*Staphylococcus aureus* -- NA

*Bacillus cereus* -- NA

*Erwinia carotovora* -- NA

**Fungal species**

*Fusarium oxysporum* ++ 18.30±0.58

*Fusarium moniliforme* ++ 14.80±0.33

*Fusarium graminearum* -- NA

*Aspergillus flavus* + 16.92±0.39

*Colletotrichum caspasci* -- NA

*Candida albicans* -- NA

*Penicillium citrium* ++ 15.70±0.84

-- negative, ++ good; ±denote standard deviation; NA no activity
