# Supplementary material for: ACC deaminase producing rhizobacterium Enterobacter cloacae ZNP-4 enhance abiotic stress tolerance in wheat plant
Source: PLoS One. 2022 May 6;17(5):e0267127. doi: 10.1371/journal.pone.0267127 (PMC9075627; doi:10.1371/journal.pone.0267127)
Supplement: S2 Table — (DOCX) [file pone.0267127.s002.docx]

**S2 Table Physiochemical properties of soil**

**Parameter Value**

pH 7.32±0.05

EC 0.138±0.03ds m^-1^

Olsen P 31.5 ±1.1mg kg^-1^

Total N 66 ±2.2 mg kg^-1^

Total K 121.0 ±2.6 mg kg^-1^

Zn 0.209 ±0.003mg kg^-1^

Cu 0.139 ±0.003mg kg^-1^

Fe 2.48 ±0.05 mg kg^-1^

Mn 0.947 ±0.04 mg kg^-1^
